# Teplizumab in Stage 2 Type 1 Diabetes: Clinical Practice Experience on Feasibility in 3 Adult Patients

**DOI:** 10.1111/dom.70626

**Published:** 2026-03-08

**Authors:** V. Guarnotta, F. Pantò, E. Vigneri, G. Piombo, M. I. Mineo, L. Tomasello, G. Arnaldi

**Affiliations:** ^1^ Unit of Endocrinology, Department of Health Promotion, Mother and Child Care, Internal Medicine and Medical Specialties Policlinico Paolo Giaccone, Università degli Studi di Palermo Palermo Italy

**Keywords:** dysglycaemia, immunotherapy, polyglandular autoimmune syndrome, thyroid autoimmune disease

## Abstract

**Aim:**

To describe the first Italian experience with teplizumab administered under a compassionate use programme for adults with Stage 2 type 1 diabetes (T1D), and to evaluate its feasibility, safety and early metabolic outcomes in a real‐life clinical setting outside formal clinical trials.

**Materials and Methods:**

Three adult women (aged 20–40 years) with Stage 2 T1D, identified through islet autoantibody screening and dysglycaemia assessment, received a 14‐day intravenous course of teplizumab (Tzield) in a day‐hospital setting. Infusions were managed by a multidisciplinary team with standardised pre‐medication and monitoring. Clinical and laboratory parameters (vital signs, lymphocyte count, liver enzymes, HbA1c, C‐peptide, continuous glucose monitoring metrics) were recorded at baseline and after 3 months.

**Results:**

Treatment was overall well tolerated. One patient developed mild transient elevation of transaminases and lymphopenia, resolving spontaneously. A second patient experienced a 40% reduction in lymphocyte count without symptoms. The third developed a mild cytokine‐release syndrome on Day 5, which resolved with supportive therapy but led to treatment discontinuation due to anxiety. At 3‐month follow‐up, all patients maintained stable glycaemic control and preserved β‐cell function.

**Conclusions:**

Teplizumab administration under compassionate use proved feasible and safe in a real‐life academic context, consistent with pivotal trial data. The experience underscores the importance of a structured multidisciplinary approach, integrating endocrinologists, anaesthesiologists, nurses and psychologists to optimise adherence, safety and patient engagement during early disease‐modifying immunotherapy in presymptomatic T1D.

## 1 Introduction

T1D is a complex, multifactorial disease resulting from the interaction between genetic susceptibility, environmental exposures, pancreatic β‐cell vulnerability and immune‐mediated mechanisms [1]. Genetic risk is largely determined by HLA class II haplotypes and multiple non‐HLA loci, which together influence immune regulation and β‐cell resilience. Environmental factors, including viral infections, nutritional patterns, gut microbiota composition and metabolic stress, may act as triggers or accelerators of β‐cell‐directed autoimmunity in genetically predisposed individuals.

The autoimmune process involves both humoral and cellular components, characterised by the development of multiple islet autoantibodies and the progressive infiltration of pancreatic islets by immune cells. Although CD8^+^ T lymphocytes play a central role in mediating β‐cell destruction, CD4^+^ T cells, B lymphocytes, macrophages and innate immune pathways also contribute to disease progression. In parallel, intrinsic β‐cell stress responses, endoplasmic reticulum dysfunction and altered proinsulin processing may enhance immunogenicity and susceptibility to immune attack.

The natural history of T1D unfolds gradually through four well‐defined stages, which reflect the progression from an early autoimmune phase to overt clinical disease. The disease progresses for years after seroconversion, resulting in the loss of most insulin‐producing β‐cells [2, 3]. In the early phase (Stage 1), individuals show two or more islet autoantibodies indicating active β‐cell autoimmunity, but glucose levels remain normal and no metabolic changes are detectable. In Stage 2, the autoimmune process progresses and mild metabolic alterations appear, causing dysglycaemia while the person is still asymptomatic. This phase is divided into 2a (slightly elevated glucose) and 2b (values near diabetic thresholds) [4]. Stage 3 corresponds to the clinical onset of T1D, defined by overt hyperglycaemia (fasting plasma glucose ≥ 126 mg/dL, 2‐h plasma glucose after an oral glucose tolerance test ≥ 200 mg/dL and/or HbA1c value ≥ 6.5% in asymptomatic patients or random plasma glucose ≥ 200 mg/dL in symptomatic patients) meeting ADA criteria [5]. Some individuals (3a) are diagnosed while asymptomatic, whereas others (3b) develop the typical symptoms, polyuria, polydipsia and weight loss—requiring insulin. Stage 4 represents established, permanent T1D, with near‐complete β‐cell loss and lifelong dependence on insulin therapy [5, 6, 7].

Teplizumab (Tzield) is a humanised monoclonal antibody directed against the CD3 chain of the T‐cell receptor complex on T lymphocytes. By inducing partial agonistic signalling, it temporarily renders autoreactive T cells unresponsive to antigenic stimulation, leading them into a state of functional exhaustion characterised by reduced cytotoxic and pro‐inflammatory activity [8, 9, 10]. At the same time, teplizumab enhances immune tolerance by expanding and activating regulatory T cells, which are crucial for controlling autoreactive immune responses and protecting pancreatic β‐cells from the autoimmune destruction. Immunophenotypic analyses have provided mechanistic insights into teplizumab's immunomodulatory action. The drug selectively reduces autoreactive CD8^+^ T‐cells while preserving antiviral immunity, avoiding generalised immunosuppression. It promotes the differentiation of partially exhausted CD8^+^ T cells, characterised by reduced cytotoxic potential and expands the pool of functional regulatory T cells, resulting in durable immune tolerance.

Through this dual mechanism, attenuation of effector T‐cell activity and reinforcement of regulatory networks, teplizumab has emerged as the first therapy capable of modifying the natural history of T1D. The drug is currently approved in the United States and in several non‐EU countries for adults and paediatric patients aged 8 years or older with Stage 2 presymptomatic T1D [11, 12].

The At‐Risk (TN‐10) phase 2 randomised, placebo‐controlled trial provided the key evidence for teplizumab's clinical use [13]. In 76 high‐risk individuals aged 8–45 years with Stage 2 T1D and first‐degree relatives of T1D patients, a single 14‐day course of teplizumab significantly delayed progression to clinical diabetes. The median time to Stage 3 was 48.4 months with teplizumab versus 24.4 months with placebo, reflecting a 59% lower risk of progression. During follow‐up, 43% of treated participants developed diabetes compared with 72% in the placebo group, underscoring its clinically meaningful effect.

An extended follow‐up analysis by Sims et al. [14] further strengthened these findings, showing a median delay of 2.7 years in progression to Stage 3 disease (59.6 vs. 27.1 months) and sustained preservation of β‐cell function, as reflected by higher C‐peptide levels (1.94 vs. 1.72 pmol/mL) and improved endogenous insulin secretion in the teplizumab group.

Collectively, these findings establish teplizumab as the first disease‐modifying immunotherapy capable of delaying the onset of clinical T1D by approximately 2–3 years in high‐risk individuals, while maintaining β‐cell function and modulating autoimmunity through selective T‐cell reprogramming.

The aim of the present study is to describe the real‐world clinical implementation, feasibility and short‐term safety of teplizumab in adult patients with Stage 2 T1D in a routine clinical practice setting.

## 2 Case Presentations

We present three women (aged 20–40 years) with Stage 2 T1D treated with teplizumab under a compassionate use authorisation, (https://aemmedi.it/wp‐content/uploads/2024/12/Documento‐SID‐AMD‐USO‐COMPASSIONEVOLE‐TEPLIZUMAB_dicembre‐2024.pdf) providing real‐life clinical evidence of its feasibility and safety in routine care. Clinical consents were obtained for treatment and case presentations. Patients were identified through routine clinical follow‐up and targeted autoantibody screening. Our unit is a referral centre for rare and autoimmune endocrine diseases and has implemented structured screening programmes for the identification of individuals at high risk of developing T1D. Notably, none of the three individuals identified at our centre had a first‐degree relative affected by T1D, in contrast to the cohort described in the pivotal study [13]. One patient had a history of gestational diabetes diagnosed approximately 3 years earlier, which had resolved postpartum, and was subsequently followed at our clinic. A second patient, affected by autoimmune polyendocrinopathy, was already under follow‐up for Stage 1 T1D. The third patient was identified through autoantibody screening performed after the incidental finding of a random blood glucose level of 200 mg/dL in the context of autoimmune thyroiditis in a normal‐weight individual. All three patients tested positive for multiple islet autoantibodies (antibodies anti GAD, anti IA2, anti insulin, anti ICA and anti ZnT8) (Table 1) [15], thus meeting the diagnostic criteria for Stage 2 T1D.

**TABLE 1:** Characteristics of patients included in the study at baseline.

	Patient 1	Patient 2	Patient 3
Age (years)	20	37	40
Sex	F	F	F
BMI (kg/m^2^)	24.6	29.1	19.3
BSA (m^2^)	1.59	1.69	1.44
Method of detection	Systematic screening	Systematic screening	Systematic screening
Familial history of type 1 autoimmune diabetes	No	No	No
Other autoimmune disorders (year of diagnosis)	Autoimmune thyroiditis	Autoimmune thyroiditis and Addison disease	None
HbA1c values (%)	5.1	6	6.4
Fasting c‐peptide (mcg/L)	1.58	3.51	1.76
C‐peptide peak during mixed meal oral test (mcg/L)	7.89	8.5	7.34
Glucose during oral glucose tolerance test (0–30–60–90 and 120 min) (mg/dL)	73–167–200–196–169	78–162–142–130–103	93–191–163–104–81
Insulin during oral glucose tolerance test (0–30–60–90 and 120 min) micro U/mL	2.9–101–132–224–220	2.5–155–126–145–116	4.7–25.2–78.9–68.7–34.5
Pancreatic islet autoantibodies (Ab) positivity	anti‐IAA antiZnT8	anti‐GAD anti‐ICA	anti‐GAD anti‐ICA
Hepatitis C virus IgG	Negative	Negative	Negative
Hepatitis B virus HBsAg	Negative	Negative	Negative
Hepatitis B virus anti‐HBc Ig	Negative	Negative	Negative
Cytomegalovirus IgG	Positive	Positive	Positive
Cytomegalovirus IgM	Negative	Negative	Negative
Epstein–Barr virus IgG	Negative	Positive	Negative
Epstein–Barr virus IgM	Negative	Negative	Negative

Abbreviations: GAD65, glutamic acid decarboxylase 65 antibodies; IAA, insulin autoantibodies; ICA, islet cell autoantibodies; ZnT8, zinc transporter 8 autoantibodies.

Patient 1 was a 20‐year‐old woman who was the first to be treated with teplizumab at our centre. She had an autoimmune hypothyroidism diagnosed in 2023 and was on levo‐thyroxine treatment with a recent occasional detection of post‐prandial dysglycaemia during oral glucose tolerance test (OGTT). She had high anti ZnT8 (32 IU/mL; normal is< 15 IU/mL) and insulin autoantibody (34.8 UI/mL; normal is< 10 IU/mL), HbA1c was 5.1%. She had a familial history of autoimmune thyroid disease and type 2 diabetes mellitus.

Patient 2 was a 38‐year‐old woman who had autoimmune hypothyroidism diagnosed in 2003 and Addison's disease diagnosed in 2018. She was on treatment with levo‐thyroxine, dual‐release hydrocortisone and fludrocortisone. She was periodically followed up at our centre. She had Stage 1 T1D with a recent dysglycaemia onset. She had a high HbA1c of 6% and post‐prandial dysglycaemia during OGTT. She had high GAD (> 250 IU/mL; normal is < 5 IU/mL) and islet cell antibodies (ICA) (21.7 UI/mL, normal < 0.9). She had no familial history of autoimmune disease.

Patient 3 was a 40‐year‐old woman who had a history of gestational diabetes mellitus developed during pregnancy in 2022. After delivery she was followed up at our centre. She developed dysglycaemia after 30 months from the delivery. The antibodies were positive for GAD (> 250 UI/ml; normal < 5) and ICA (180 UI/ml; normal < 0.9). She had psoriasis and a familial history of autoimmune thyroid disease.

A Freestyle Libre 2 CGM device was applied during the infusion and after 3 months of follow‐up.

The patients started teplizumab infusion (Tzield, Sanofi), with personalised dosing, body surface area (BSA)–based dosing schedule. Tzield was administered by intravenous infusion over a minimum of 30 min once daily for 14 consecutive days, following the approved dose‐escalation regimen: Day 1: 65 μg/m^2^; Day 2: 125 μg/m^2^; Day 3: 250 μg/m^2^; Day 4: 500 μg/m^2^; Days 5–14: 1030 μg/m^2^ (https://www.accessdata.fda.gov/drugsatfda_docs/label/2022/761183s000lbl.pdf). All infusions were performed during day hospital admissions at our clinical centre. Each patient attended the unit in the morning and was monitored throughout the infusion period without overnight stay. Vital signs and laboratory parameters were assessed before, during and after the infusion, as recommended. Before initiating treatment, a dedicated team of expert anaesthesiologists (vascular team) placed a Midline catheter to ensure safe and reliable intravenous access for the entire infusion period. The nursing team, after receiving specific training on study procedures, prepared the investigational product according to the manufacturer's instructions. During the first 5 days of treatment, infusion times were intentionally prolonged, lasting at least 1.5 h, followed by an observation period of approximately 2.5 h within the hospital ward to ensure patient safety and monitor for potential infusion‐related reactions.

At the end of the observation period, patients were discharged home on the same day, with instructions for follow‐up monitoring. All patients had pre‐treatment with acetaminophen, metoclopramide and diphenhydramine for the first 5 days of infusion.

**FIGURE 1:**
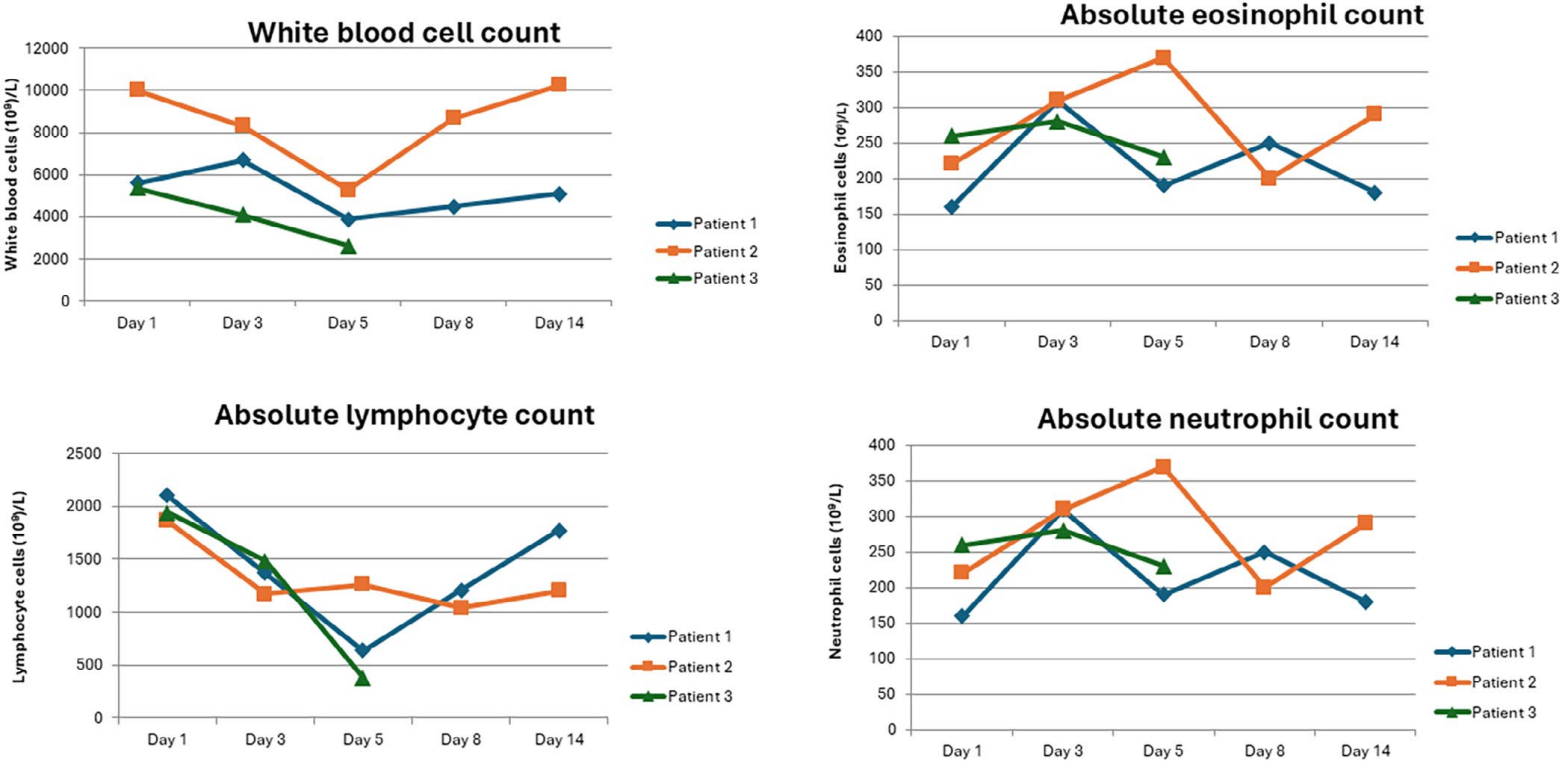
Changes in peripheral blood leukocyte counts during teplizumab treatment. White blood cell, eosinophil, lymphocyte, and neutrophil counts are shown at predefined time points (Days 1, 3, 5, 8, and 14). Values are expressed as cells/µL.

**FIGURE 2:**
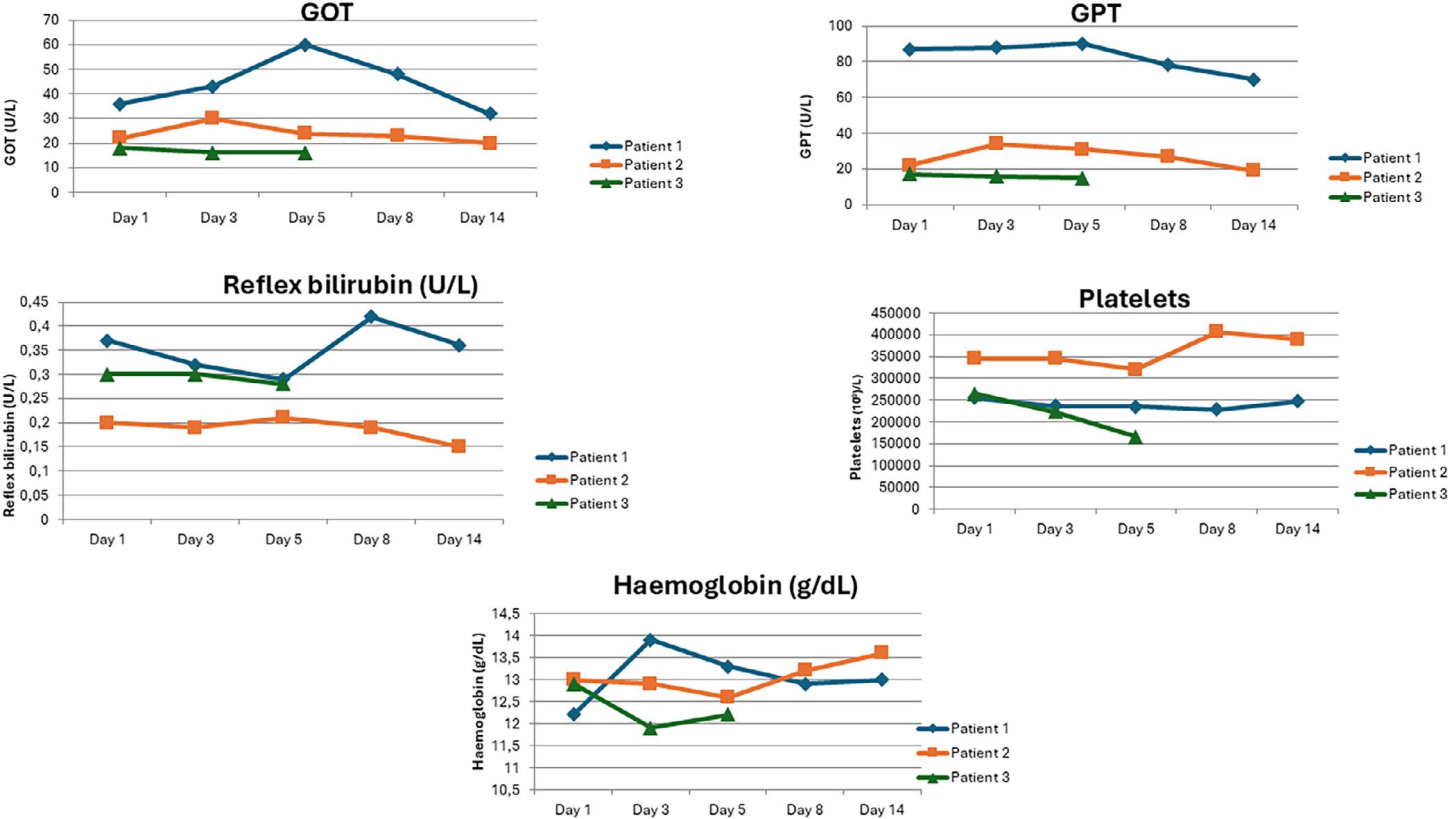
Longitudinal changes in biochemical and haematological parameters during teplizumab treatment shown for each patient at predefined time points (Days 1, 3, 5, 8, and 14).

The clinical course was different for each patient (Figures 1 and 2). During the first day of Tzield administration, patient 1 developed a mild fever (Tc 37°C) that resolved spontaneously within a few hours without pharmacological treatment. On the third day of infusion, a transient increase in liver enzymes was observed (GOT: 2 × ULN; GPT: 4 × ULN), which improved over the following days, with GOT decreasing to 1.5 × ULN and GPT to 2 × ULN by day 8 (Figure 2). On the fifth day of treatment, a reduction in lymphocyte count by almost 70% was noted (640 cells/μL), which subsequently recovered to 1200 cells/μL by Day 8 (Figure 1).

The second patient did not develop any clinical symptoms and remained clinically stable; therefore, no stress‐dose glucocorticoid replacement for adrenal insufficiency was required either during the infusion days or in the subsequent follow‐up period. She developed an approximate 40% reduction in lymphocyte count compared to baseline on the seventh day, while maintaining levels consistently above 1000 cells/μL (Figure 1).

The third patient experienced nausea on days 2 and 3 of treatment which recovered spontaneously. On day 5, approximately 6 h after interruption of the infusion, she developed a mild to moderate cytokine release syndrome characterised by high‐grade fever (> 39°C), nausea, vomiting and truncal and inguinal erythema, accompanied by a marked reduction in lymphocyte count by more than 80% (380 cells/μL) (Figure 1). The patient was treated with acetaminophen, metoclopramide and ebastine and teplizumab infusion was discontinued. On Day 6, interleukin‐6 and C‐reactive protein levels were assessed and found to be elevated. Intravenous hydration was administered, resulting in an improvement of symptoms. On Day 7, truncal erythema persisted but was slightly improved; no fever or vomiting was reported. A second intravenous hydration was administered. On Day 8, topical corticosteroid therapy was initiated for the erythema. By Day 8, the lymphocyte count had increased to 680 cells/μL (Figure 1). By Day 10, clinical symptoms had completely resolved and lymphocyte levels had returned to normal. However, the treatment was definitely interrupted due to the patient's persistent anxiety.

At 3‐months of follow‐up patient 1 showed a clear decline of IA2 antibodies, while ZnT8 antibody levels increased, and the remaining autoantibodies stayed negative (Table 2). From a metabolic standpoint, glucose control remained stable as evaluated by HbA1c, glucose control monitoring, as well as for c‐peptide concentrations as a marker of endogenous β‐cell secretory function.

**TABLE 2:** Follow‐up in the two patients who completed Teplizumab infusion course.

	Patient 1	Patient 2	Patient 3
GAD antibodies (UI/mL)			
Baseline	< 1	> 250	> 250
3 months	< 1	> 250	> 250
IA2 antibodies (UI/mL)			
Baseline	34.8	< 1	< 1
3 months	19.2	< 1	< 1
ZnT8 antibodies (UI/mL)			
Baseline	32.09	0.01	< 0.01
3 months	66.2	0.1	0.1
ICA antibodies (UI/mL)			
Baseline	< 1	21.7	180
3 months	< 1	19.1	179
Insulin antibodies (UI/mL)			
Baseline	< 1	0.3	1.7
3 months	0.3	0.2	1.7
HbA1c (%)			
Baseline	5.1	6	6.4
3 months	5.4	5.8	6.3
Fasting glucose values (mg/dL)			
Baseline	73	78	105
3 months	72	99	103
Mean Postprandial glucose (mg/dL)			
Baseline	165	162	168
3 months	101	114	92
Time above range (> 140 mg/dL) (%)			
During infusion	1	1	2
3 months	0	0	2
Time below range (< 70 mg/dL) (%)			
During infusion	0	0	1
3 months	2	0	0
Time in range (70–140 mg/dL) (%)			
During infusion	99	99	97
3 months	98	100	98
Fasting C‐peptide value (mcg/L)			
Baseline	1.58	4.51	1.76
3 months	1.75	3.88	1.84
Peak stimulated C‐peptide (mcg/L)			
Baseline	7.89	8.5	6.37
3 months	8.2	7.9	7.05

In Patient 2, GAD antibody titers remained markedly elevated (> 250 UI/mL) and ICA levels showed a slight decrease from 21.7 to 19.1 UI/mL (Table 2). No differences in other pancreas autoantibodies. Glycaemic control remained excellent during follow‐up, with continuous glucose monitoring metrics showing optimal stability (Table 2). C‐peptide levels displayed a mild decline over time, from 4.51 to 3.88 μg/L in the fasting state and from 8.5 to 7.9 μg/L after stimulation (Table 2). Despite this modest reduction, both values remained well within the physiological range, indicating preserved β‐cell secretory capacity and sustained metabolic stability.

In Patient 3, GAD and ICA autoantibody titers remained markedly elevated (> 250 and 179 UI/mL, respectively) (Table 2). No relevant changes were observed in other pancreatic autoantibodies, with IA‐2 antibodies remaining negative and only minimal ZnT8 positivity detected. From a metabolic perspective, glycaemic control remained stable, as reflected by HbA1c, fasting glucose values and continuous glucose monitoring metrics, which showed optimal stability throughout follow‐up (Table 2). C‐peptide concentrations were stable, indicating preserved endogenous β‐cell secretory function at 3 months despite the reduced duration of infusion.

## 3 Discussion

This case series reports the first Italian real‐life experience of teplizumab treatment on compassionate use in three adult patients with Stage 2 T1D. All three women met the diagnostic criteria for Stage 2 disease and received teplizumab according to the approved 14‐day regimen. Treatment was well tolerated overall, with manageable infusion reactions and no serious adverse events in the first two patients.

Our findings are consistent with data from pivotal clinical trials, particularly the At‐Risk (TN‐10) study and its long‐term follow‐up, which established teplizumab as the first disease‐modifying immunotherapy capable of delaying progression from Stage 2 to Stage 3 T1D by 2–3 years. In both controlled and real‐world settings, transient lymphopenia and mild transaminase elevations have been the most frequent laboratory findings, reflecting expected pharmacodynamic effects of CD3 modulation.

Recently, Wilson et al. [16] reported the use of teplizumab in two adolescents with autoimmune polyendocrine syndrome type 1 (APS‐1) and Stage 2 type 1 diabetes, providing an important comparison in the context of complex multisystem autoimmunity. Similar to our study, in this real‐world clinical experience, the short‐term feasibility and safety of anti‐CD3 therapy outside controlled trial settings was reported. Notably, in both cohorts no clinically relevant deterioration of concomitant autoimmune diseases was observed during treatment, and extra‐pancreatic manifestations remained stable. Indeed, thyroid and adrenal hormone replacement regimens remained unchanged during follow‐up, supporting the short‐term endocrine safety of treatment.

Further support for the clinical relevance of real‐world teplizumab use is provided by the recent prospective observational study by Karakus et al. [17], which represents the first systematic evaluation of teplizumab following regulatory approval in individuals with Stage 2 T1D. In that study, glycaemic responses observed in pivotal trials were largely reproduced in routine clinical practice, and early immunological changes, including reductions in CD4 preproinsulin‐specific T‐cell receptors, were identified as potential biomarkers of treatment response. These findings suggest that post‐treatment immune profiling may help refine early‐stage therapeutic strategies and patient selection.

In parallel, a recent comprehensive review by Mathieu et al. [18] has placed teplizumab within the evolving landscape of disease‐modifying therapies for T1D, highlighting its role as the first approved immunomodulatory treatment capable of delaying clinical onset. Together, these data support the progressive integration of teplizumab into standard care pathways and reinforce the clinical relevance of real‐world experiences, such as ours, in informing future implementation strategies.

Real‐world evidence from the RESIST‐T1D study [19] has further expanded understanding of teplizumab's clinical and psychosocial impact. Beyond the controlled setting of clinical trials, teplizumab has been generally well accepted by patients and caregivers, who viewed treatment as a proactive measure to delay disease onset and improve long‐term management. However, persistent anxiety about disease progression remains common, reflecting the psychological burden of living with an early autoimmune condition.

In our experience, starting teplizumab therapy represented not only a medical but also an emotional challenge for patients and their families. Despite extensive counselling, expectations were sometimes shaped by the perception of teplizumab as a preventive rather than disease‐delaying therapy, emphasising the need for clear communication about treatment goals. Each patient received detailed education on the drug's mechanism, benefits and risks, jointly provided by physicians and trained nursing staff to foster trust and adherence. The third patient, who presented with high baseline anxiety, experienced a mild cytokine release syndrome that resolved promptly but led to discontinuation due to persistent fear of adverse events. This case underscores how emotional factors can influence adherence, highlighting the importance of psychological assessment and continuous support during therapy.

As reported in the RESIST‐T1D study [19] and other clinical experiences [20, 21], successful implementation of teplizumab requires not only clinical expertise but also a structured, multidisciplinary care model. Both our experience and RESIST‐T1D confirm the real‐world feasibility of teplizumab administration and highlight the key role of patient anxiety and emotional burden during treatment, underscoring the importance of dedicated psychological and educational support.

Notably, while the RESIST‐T1D study, conducted as a manufacturer‐funded patient survey, provides valuable insights into patient perspectives and quality‐of‐life aspects, it lacks systematic laboratory and metabolic outcome data. Conversely, our study does not include structured patient‐reported measures of quality of life but offers detailed clinical and biochemical characterisation of treated individuals. Taken together, these two experiences should be regarded as highly complementary, bridging patient‐centred perspectives with objective clinical and laboratory outcomes in the real‐world setting.

Consistent with this multidisciplinary framework, in our centre close collaboration between endocrinologists, anaesthesiologists, psychologists and diabetes nurse educators was crucial. Anaesthesiologists ensured procedural safety through midline catheter placement and infusion monitoring, while psychologists and nurse educators addressed treatment‐related anxiety, facilitated communication and supported patient engagement. This integrated approach proved essential for maintaining adherence, safety and patient confidence throughout treatment.

As screening for early‐stage T1D becomes more widespread, especially among individuals with a family history of autoimmune disease, integrating teplizumab into structured care pathways, combining early identification, tailored education and psychological support, will be critical to optimise both clinical and emotional outcomes. In this context, emerging population‐based and targeted screening programmes, such as Fr1Da in Germany [22] and ELSA in the United Kingdom [23], have demonstrated the feasibility of large‐scale identification of individuals at early stages of T1D. As these initiatives expand, increasing numbers of presymptomatic patients are expected to be diagnosed, highlighting the need for standardised and reproducible clinical pathways for immunomodulatory interventions.

Our real‐world experience provides practical insights into how teplizumab can be implemented within routine clinical practice, complementing large screening programmes and supporting the transition from early diagnosis to personalised disease‐modifying treatment.

Accordingly, an accurate immunological profiling is essential for appropriate staging and patient selection. In line with current international consensus and ADA Standards of Care [24, 25], clinical staging and eligibility for teplizumab in our cohort were primarily based on the assessment of biochemical autoantibodies against the major pancreatic autoantigens, including IAA, GAD, IA‐2 and ZnT8. Although islet cell autoantibodies detected by indirect immunofluorescence are less commonly used in routine practice and their antigenic targets are not fully characterised, ICA testing provided additional clinically relevant information in our series. In two of three patients, ICA positivity contributed to the identification of Stage 2 disease when interpreted alongside dysglycaemia and multiple biochemical autoantibodies. Therefore, while biochemical markers remain central to disease staging, ICA assessment may still offer complementary value in selected clinical and research settings.

Interestingly, Delgado et al. [26] reported that Epstein–Barr virus (EBV) seropositivity may modulate the immunological response to teplizumab. EBV‐seropositive individuals exhibited an expansion of regulatory and partially exhausted CD8^+^ T‐cell populations, with attenuation of proinflammatory signalling pathways. As anti‐CD3 therapy similarly promotes controlled T‐cell exhaustion and immune tolerance, a pre‐existing exhaustion‐prone immune profile in EBV‐seropositive individuals may facilitate the biological effects of teplizumab. This immunological background may contribute to inter‐individual variability in treatment response and provides a plausible mechanistic basis for the potential relevance of viral profiling in future personalised therapeutic strategies.

In our cohort, only patient 2 was EBV‐seropositive and showed improved glycaemic parameters at three‐month follow‐up without additional adverse events. Characterising viral profiles prior to treatment could potentially help identify immunological phenotypes more responsive to teplizumab or predict differential immune dynamics during therapy. However, given the limited sample size and short duration of follow‐up, no definitive conclusions can be drawn regarding the impact of viral–immune interactions on β‐cell preservation. Future prospective studies with longer follow‐up and systematic viral profiling are warranted to clarify whether latent viral–immune interactions may represent a relevant modifier of treatment response.

After 3 months, the patients showed stable glycaemic control and preserved β‐cell function, consistent with previous pivotal studies demonstrating the immunomodulatory and metabolic effects of teplizumab. Nevertheless, longer follow‐up will be required to fully assess the durability of these benefits over time.

Although our short‐term observations are reassuring in terms of feasibility and tolerability, longer follow‐up is required to determine whether early immunomodulation translates into sustained clinical benefit. In Stage 2 T1D, the primary therapeutic goal is delay of progression to Stage 3 disease, an outcome that can only be robustly evaluated over years rather than months, given the variable natural history and the staged trajectory of dysglycaemia. Moreover, the durability of teplizumab's effect likely depends on the persistence of immunological reprogramming and its interaction with ongoing environmental and metabolic stressors over time. Consistent with this, the TN‐10 trial [13] and subsequent follow‐up studies [27, 28] have shown that teplizumab can confer clinically meaningful delay in progression beyond 2–3 years in subsets of individuals. Mechanistically, teplizumab has been shown to induce persistent changes in the antigen‐specific immune repertoire in at‐risk individuals, supporting the biological plausibility of longer‐term benefit that may not be captured by early metabolic endpoints alone. In addition, a recent meta‐analysis of randomised controlled trials further supports the efficacy of teplizumab in T1D across clinical settings. Accordingly, extended follow‐up with longitudinal metabolic assessment (including C‐peptide trajectories where applicable) and immunophenotyping is essential to clarify the durability of response and to identify clinical and immunological predictors of sustained benefit.

The main limitations of this report include the small sample size, short follow‐up and absence of longitudinal c‐peptide data. Nevertheless, this compassionate use–based real‐life experience offers meaningful insights into the practical, psychological and organisational challenges associated with implementing disease‐modifying immunotherapy for T1D beyond clinical trial settings, even though patient reported measures outcomes data were not recorded in the current case series. Importantly, our observations add novel safety data, as teplizumab was well tolerated even in a patient with concomitant autoimmune Addison's disease, a condition known to complicate clinical management. To our knowledge, this represents the first European real‐world report of teplizumab use in adult patients. Most available evidence to date has focused on paediatric and adolescent populations, and data in adults remain limited. In addition, our series includes individuals with APS, including adrenal insufficiency, conditions that have been only marginally represented in pivotal clinical trials. Together, these features extend current evidence on teplizumab use to complex adult autoimmune settings encountered in routine clinical practice.

In conclusion, teplizumab administration in a real‐life academic context proved feasible and safe, and especially effective when supported by a coordinated multidisciplinary team. Our findings primarily emphasise the favourable short‐term safety and tolerability profile of teplizumab, even in complex autoimmune patients managed in routine clinical practice.

## Author Contributions

V.G. made substantial contributions to the conception and design of the study; contributed to patient selection, data acquisition, analysis and interpretation of the data; drafted the manuscript; critically revised it for important intellectual content; and approved the final version to be published. F.P. made substantial contributions to data acquisition, clinical management of the patients and interpretation of the data; critically revised the manuscript for important intellectual content and approved the final version to be published. E.V. made substantial contributions to the conception and design of the study and to interpretation of the data; critically revised the manuscript for important intellectual content and approved the final version to be published. G.P. made substantial contributions to data acquisition and interpretation of the data; critically revised the manuscript for important intellectual content and approved the final version to be published. L.T. made substantial contributions to patient management, data acquisition and interpretation of the data; critically revised the manuscript for important intellectual content and approved the final version to be published. M.I.M. made substantial contributions to data acquisition and interpretation of the data; critically revised the manuscript for important intellectual content; and approved the final version to be published. G.A. made substantial contributions to the conception and design of the study and interpretation of the data; critically revised the manuscript for important intellectual content and approved the final version to be published.

## Funding

The authors have nothing to report.

## Conflicts of Interest

V.G. has received fees from Recordati. E.V. has received a fee from Sanofi for a moderation. G.A. has received fees from Lilly, Novo Nordisk. F.P., G.P., L.T. and. M.I.M. have nothing to disclose. The product was provided free of charge via Sanofi's Managed Access Program IPR0054. Sanofi was not involved in the design, collection, analysis, interpretation or reporting of the data, but was provided the opportunity to review the publication prior to submission. The decision to submit for publication was made independently by the authors.

## Data Availability

All data supporting the findings of this study are available within the paper.

## References

[1] K. I. Aamodt and A. C. Powers , “The Pathophysiology, Presentation and Classification of Type 1 Diabetes,” Diabetes, Obesity & Metabolism 27, no. S6 (2025): 15–27. 10.1111/dom.16628 PMC12312824 40734585

[2] A. Pugliese , “Insulitis in the Pathogenesis of Type 1 Diabetes,” Pediatric Diabetes 17, no. S22 (2016): 31–36. 27411434 10.1111/pedi.12388 PMC4948864

[3] A. C. Powers , “Type 1 Diabetes Mellitus: Much Progress, Many Opportunities,” Journal of Clinical Investigation 131, no. 8 (April 2021): e142242. 33759815 10.1172/JCI142242 PMC8262558

[4] A. Galderisi , E. K. Sims , C. Evans‐Molina , et al., “Trajectory of Beta Cell Function and Insulin Clearance in Stage 2 Type 1 Diabetes: Natural History and Response to Teplizumab,” Diabetologia 68, no. 3 (March 2025): 646–661. 39560746 10.1007/s00125-024-06323-0 PMC11832608

[5] N. A. ElSayed , G. Aleppo , V. R. Aroda , et al., “2. Classification and Diagnosis of Diabetes: Standards of Care in,” Diabetes Care 46, no. Supplement_1 (January 2023): S19–S40. 36507649 10.2337/dc23-S002 PMC9810477

[6] R. A. Insel , J. L. Dunne , M. A. Atkinson , et al., “Staging Presymptomatic Type 1 Diabetes: A Scientific Statement of JDRF, the Endocrine Society, and the American Diabetes Association,” Diabetes Care 38, no. 10 (October 2015): 1964–1974. 26404926 10.2337/dc15-1419 PMC5321245

[7] R. Mallone , E. Bismuth , C. Thivolet , et al., “Screening and Care for Preclinical Stage 1–2 Type 1 Diabetes in First‐Degree Relatives: French Expert Position Statement,” Diabetes & Metabolism 51, no. 1 (2025): 101603. 39675522 10.1016/j.diabet.2024.101603

[8] E. L. Ramos , C. M. Dayan , L. Chatenoud , et al., “Teplizumab and β‐Cell Function in Newly Diagnosed Type 1 Diabetes,” New England Journal of Medicine 389, no. 23 (December 2023): 2151–2161. 37861217 10.1056/NEJMoa2308743

[9] L. Chatenoud , E. Thervet , J. Primo , and J. F. Bach , “Anti‐CD3 Antibody Induces Long‐Term Remission of Overt Autoimmunity in Nonobese Diabetic Mice,” Proceedings of the National Academy of Sciences 91, no. 1 (January 1994): 123–127. 10.1073/pnas.91.1.123 PMC42898 8278351

[10] C. Kuhn and H. L. Weiner , “Therapeutic Anti‐Cd3 Monoclonal Antibodies: From Bench to Bedside,” Immunotherapy 8, no. 8 (July 2016): 889–906. 27161438 10.2217/imt-2016-0049

[11] D. Beran , C. Abidha , A. Adler , et al., “Teplizumab Approval for Type 1 Diabetes in the USA,” Lancet Diabetes & Endocrinology 11, no. 2 (2023): 78–80. 36623522 10.1016/S2213-8587(22)00384-9

[12] C. Evans‐Molina and R. A. Oram , “Teplizumab Approval for Type 1 Diabetes in the USA,” Lancet Diabetes and Endocrinology 11, no. 2 (February 2023): 76–77. 36623518 10.1016/S2213-8587(22)00390-4

[13] K. C. Herold , B. N. Bundy , S. A. Long , et al., “An Anti‐CD3 Antibody, Teplizumab, in Relatives at Risk for Type 1 Diabetes,” New England Journal of Medicine 381, no. 7 (August 2019): 603–613. 31180194 10.1056/NEJMoa1902226 PMC6776880

[14] E. K. Sims , S. M. Geyer , S. A. Long , and K. C. Herold , “High Proinsulin: C‐Peptide Ratio Identifies Individuals With Stage 2 Type 1 Diabetes at High Risk for Progression to Clinical Diagnosis and Responses to Teplizumab Treatment,” Diabetologia 66, no. 12 (December 2023): 2283–2291. 37667106 10.1007/s00125-023-06003-5 PMC10914155

[15] S. B. Leichter , J. L. Felton , C. Geno Rasmussen , et al., “Establishing Screening Programs for Presymptomatic Type 1 Diabetes: Practical Guidance for Diabetes Care Providers,” Journal of Clinical Endocrinology and Metabolism 110, no. 8 (July 2025): 2371–2382. 40171881 10.1210/clinem/dgaf194 PMC12261085

[16] C. S. Wilson , A. Falk , J. M. Williams , et al., “Use of Teplizumab to Modulate Stage 2 Type 1 Diabetes in Two Individuals With Autoimmune Polyendocrine Syndrome 1,” Diabetes Care 49, no. 1 (January 2026): 111–117. 41223156 10.2337/dc25-1444 PMC12719717

[17] K. E. Karakus , L. Chesshir , S. Walker , et al., “Teplizumab Treatment for Stage 2 Type 1 Diabetes: A Real‐World Evaluation of Metabolic and Immunological Outcomes,” Diabetologia (2026): 1–12, 10.1007/s00125-025-06646-6. 41535597 PMC13005873

[18] C. Mathieu , E. K. Sims , L. Chatenoud , E. A. James , M. A. Atkinson , and K. C. Herold , “Toward Disease‐Modifying Therapies in Type 1 Diabetes: Focus on Teplizumab,” Diabetes Care (November 2025): dci250066. 10.2337/dci25-0066 PMC12925991 41196630

[19] H. K. O'Donnell , K. M. Simmons , S. E. Gitelman , et al., “Real‐World Experiences of Adult Individuals or Caregivers of Children Who Received Teplizumab Treatment in Stage 2 Type 1 Diabetes,” Diabetes, Obesity & Metabolism 27, no. 5 (May 2025): 2495–2506. 10.1111/dom.16246 PMC11965029 39949173

[20] C. J. Duckworth , R. J. Kreienkamp , E. C. Rieger , T. H. Nguyen , J. L. Gaglia , and B. S. Lennerz , “An Atypical Presentation of Cytokine Release Syndrome With Signs of Arthritis During Treatment With Teplizumab in a Pediatric Patient,” Diabetes Care 48, no. 4 (April 2025): e49–e50. 39841557 10.2337/dc24-2322 PMC12081315

[21] J. L. Felton , A. Tuttle , and E. K. Sims , “Teplizumab‐Mzwv: Perspective on Clinical Practice and Use at a Single Institution,” SMART‐MD Journal of Precision Medicine 2, no. 2 (May 2025): e149–e157.

[22] J. Raab , F. Haupt , M. Scholz , et al., “Capillary Blood Islet Autoantibody Screening for Identifying Pre‐Type 1 Diabetes in the General Population: Design and Initial Results of the Fr1da Study,” BMJ Open 6, no. 5 (May 2016): e011144. 10.1136/bmjopen-2016-011144 PMC4874167 27194320

[23] L. M. Quinn , J. Elliott , T. Papanikolaou , et al., “Feasibility of General Population Screening for Type 1 Diabetes in the UK: The ELSA Study,” Lancet Diabetes & Endocrinology 14 (2026): S2213858725003638. 10.1016/S2213-8587(25)00363-8 41576975

[24] M. Phillip , P. Achenbach , A. Addala , et al., “Consensus Guidance for Monitoring Individuals With Islet Autoantibody–Positive Pre‐Stage 3 Type 1 Diabetes,” Diabetes Care 47, no. 8 (June 2024): dci240042. 10.2337/dci24-0042 PMC11381572 38912694

[25] M. Phillip , P. Achenbach , A. Addala , et al., “Consensus Guidance for Monitoring Individuals With Islet Autoantibody‐Positive Pre‐Stage 3 Type 1 Diabetes,” Diabetologia 67, no. 9 (September 2024): 1731–1759. 38910151 10.1007/s00125-024-06205-5 PMC11410955

[26] A. Lledó‐Delgado , P. Preston‐Hurlburt , L. Higdon , et al., “Latent EBV Enhances the Efficacy of Anti‐CD3 mAb in Type 1 Diabetes,” Nature Communications 16, no. 1 (May 2025): 5033. 10.1038/s41467-025-60276-5 PMC12125364 40447640

[27] A. Lledó‐Delgado , P. Preston‐Hurlburt , S. Currie , et al., “Teplizumab Induces Persistent Changes in the Antigen‐Specific Repertoire in Individuals at Risk for Type 1 Diabetes,” Journal of Clinical Investigation 134, no. 18 (September 2024): e177492. 39137044 10.1172/JCI177492 PMC11405034

[28] E. Heidari , A. Shafiee , S. Noorian , et al., “Efficacy of Teplizumab for Treatment of Type 1 Diabetes: A Meta‐Analysis of Randomized Controlled Trials,” Diabetes/Metabolism Research and Reviews 40, no. 4 (May 2024): e3806. 38757421 10.1002/dmrr.3806

